# Strength and Durability Characterization of Structural Concrete Made of Recycled Plastic

**DOI:** 10.3390/ma17081841

**Published:** 2024-04-17

**Authors:** Jonathan Oti, Blessing O. Adeleke, Mihiri Rathnayake, John M. Kinuthia, Emma Ekwulo

**Affiliations:** 1 Faculty of Computing, Engineering and Science, University of South Wales, Pontypridd CF37 1DL, UK; jonathan.oti@southwales.ac.uk (J.O.); mihirinr@gmail.com (M.R.); john.kinuthia@southwales.ac.uk (J.M.K.); 2 Department of Civil Engineering, Faculty of Engineering, Rivers State University, Porth Harcourt PMB 5080, Rivers State, Nigeria; eoekwulo@gmail.com

**Keywords:** waste plastics, compressive strength, consistency, tensile strength, freeze and thaw

## Abstract

This study investigates the feasibility of utilizing recycled plastic waste as a partial substitute for sand in concrete production. Reprocessing used plastic items or materials involves collecting, cleaning, shredding, and melting, resulting in reprocessed plastic particles. Incorporating these recycled plastic particles into concrete addresses environmental concerns related to plastic disposal and the growing scarcity and increasing cost of natural sand. To evaluate the sand replacement capacity of recycled plastic, four types of mixtures were created with varying levels of recycled plastic replacement (5%, 10%, 15%, and 20%). All mixtures maintained a water-to-binding ratio of 0.55 and were tested at 7, 28, and 56 days. The testing regimen encompassed determining the slump value, density, compressive strength, tensile strength, and resistance to freezing and thawing. The findings revealed that replacing sand in the concrete mix with recycled plastic enhanced workability, which was attributed to the hydrophobic nature of the plastic particles. However, both compressive and tensile strength exhibited a declining trend. Additionally, after undergoing multiple freezing and thawing cycles, the concrete mix exhibited poor durability properties and brittleness. These issues may arise due to factors such as incompatibility, non-uniformity, reduced cohesion, and the lower density of plastic particles.

## 1. Introduction

Concrete is a widely used building material, prized for its strength and availability. However, in our current age, sustainable development is a top priority. Traditional concrete production relies on basic building components like granite, sand, and cement. Cement manufacturing is energy-intensive and relies on naturally occurring materials such as shale and limestone, which may become scarce in the future [1]. Furthermore, the scarcity of sand is on the rise due to its use in manufacturing glass, concrete, and electronics. Sand shortages disrupt ecosystems and drive up the cost of domestically sourced sand, adding to environmental concerns [1]. Therefore, many researchers and technologists have been challenged to seek and develop innovative materials that rely on renewable resources in response to growing concerns about resource depletion and environmental pollution.

One such approach involves using residues and waste products for new construction purposes. A significant portion of these remnants is being repurposed as aggregates to create lightweight concrete [2]. Civil engineers have been tasked with transforming industrial by-products into useful construction materials to meet the high demand in the construction industry. This demand has intensified in recent years due to population growth, which has led to a persistent shortage of building materials [2]. Steyn et al. [3] conducted experiments to assess the impact of substituting plastic for concrete in terms of compressive strength. Their study examined how increasing the proportions of angular waste plastic particles affected the cylinder strength. This investigation was conducted across three distinct water-to-binder ratios. The results of this study revealed a decrease in compressive strength as the content of plastic aggregates increased. This reduction in strength was attributed to the inadequate bonding between the plastic particles and the cement paste in the concrete mixture.

In a study conducted by Saikia and de Brito [4], various concrete mixtures were tested, each containing three categories of differently sized and shaped particles: (1) Large particles with lengths ranging from 10 to 20 mm. (2) Shredded, flaky fine particles measuring 2 to 5 mm in length. (3) Cylindrical pellet-shaped particles measuring 3 mm in length. Each of these concrete mixtures was subjected to testing with varying replacement ratios, ranging from 0% to 15% of the sand component. The results of the study revealed that as the replacement ratio increased, the compressive strength of the concrete decreased. This decrease in strength was attributed to a lack of effective interaction between the PET aggregate and the cement paste in the concrete mix. The research also concluded that the interfacial transition zone in concrete containing PET aggregates proved weaker than in standard concrete. A study conducted by Albano et al. [5] involved the use of irregularly shaped PET particles ranging in size from 2.6 mm to 11.4 mm. These particles were incorporated as replacements at 10% and 20% proportions, and two different water-to-cement ratios were used (0.50 and 0.60). The study’s findings indicated that as the proportion of plastic increased, the compressive strength of the concrete decreased. This decrease in strength suggested that the plastic particles acted as structural defects within the concrete. Interestingly, mix designs exclusively featuring larger plastic particles were notably weaker when compared to those containing only smaller PET particles. The presence of a honeycomb-like structure composed of cavities and pores was observed in both large and small plastic particles, which was associated with low workability and had an adverse impact on concrete compaction.

Frigione [6] conducted a study in which granulated PET closely matched the grading of the siliceous sand intended for replacement in the concrete mixture. The results of this study demonstrated that, although there was a decrease in the compressive strength of the mix, the reduction was minimal, measuring less than 2%, when employing a 5% replacement ratio. This is in contrast to the 12% loss reported by Saikia and de Brito [4] when replacing 5% of sand with larger plastic pellets. These findings suggest that while the use of plastic in concrete may lead to a reduction in compressive strength due to a weaker bond with the surrounding matrix compared to sand, the extent of this loss can be effectively mitigated through careful mix design and appropriate plastic selection. The research conducted by Albano et al. [5] demonstrated that both larger plastic particles and higher replacement percentages led to significant reductions in tensile strength in concrete due to increased voids within the material. This finding was supported by Frigione [6], where a 5% replacement of sand with granulated PET resulted in only a 2% decrease in tensile strength. Similarly, Saikia and de Brito [4] observed a reduction in tensile performance as plastic aggregates were introduced into concrete, with a more pronounced loss at higher plastic content. Microscopic examinations of failed specimens revealed that the most common form of failure involved debonding at the plastic–concrete interface. Ferreira et al. [7] explored the influence of three different curing conditions on concrete with plastic waste aggregates’ mechanical performance and found that the dominant factor affecting performance was not the curing conditions but the replacement percentage. Safi et al. [8] examined the use of waste plastic bags in the production of self-compacting mortar mixes. They observed reductions in strength proportionate to the replacement percentage, with a 15% average strength reduction at 30% substitution. These reductions in strength were attributed to poor bonding between the plastic and the surrounding cement paste, aligning with previous research findings.

Most literature review studies have focused on the effect of adding plastic wastes on the workability and compressive strength (the ability of a material to resist the direct pressure of applied compression force), splitting tensile strength (an indirect way of evaluating the tensile test of concrete), and flexural strength of concrete (stress at failure in bending), with less emphasis on the durability characterization of the concrete. In this context, the present article took a step further by characterizing the durability properties of the concrete made with recycled plastic, by subjecting the test specimens to freezing and thawing cycles. The replacement of fine aggregate (sand) with recycled plastics will help to reduce the environmental concerns arising from the over-dredging of sand, which have led to restrictions on its extraction worldwide, with direct economic impacts on concrete production. Recycled plastic aggregates can also be used for producing concrete bricks (for general applications), blocks (for river bank protection), façade elements, non-structural concrete panels, and temporary shelters. For structural concrete applications, structures with lower imposed loads and where durability is less important, a certain amount of plastic aggregates may be used in concrete. Plastic fibers can be used in concrete to control cracks, shrinkage, and creep rather than using expensive synthetic or steel fibers. This will reduce not only the dependence on the natural aggregates but possibly also the cost of concrete.

## 2. Materials and Methods

**Figure 1:**
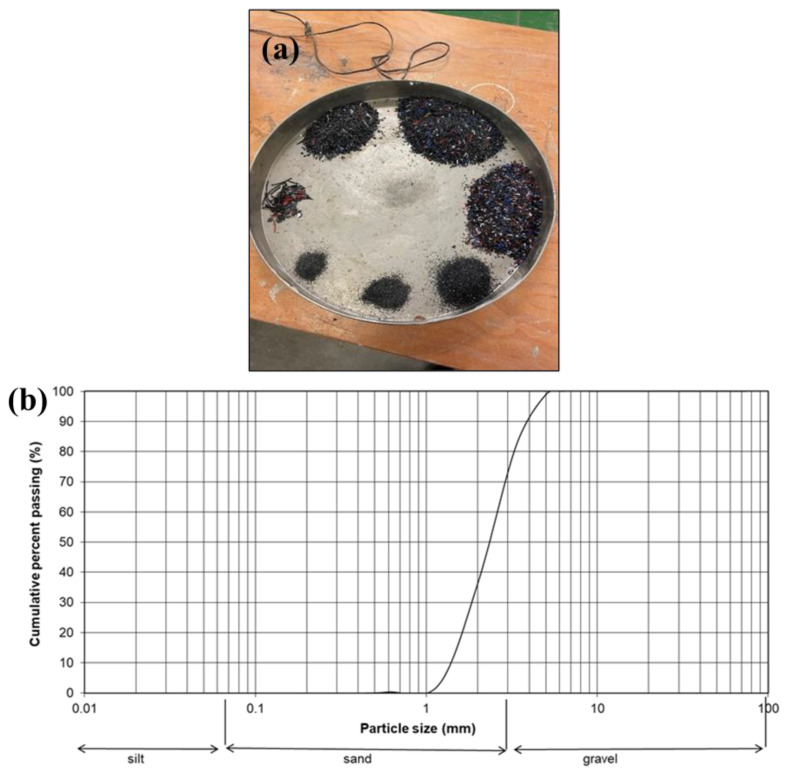
(**a**) Sieve analysis samples, and (**b**) particle size distribution of recycled plastics.

The materials used in this study are ordinary Portland cement, coarse aggregate, sand, recycled plastic, and water. The Portland cement used in this study was sourced from Lafarge Cement (Barnstone, UK) and has a minimum strength of 32.5 N/mm². Coarse aggregates consisted of limestone with particle sizes of 10 mm and 20 mm. Natural sea-dredged sand from the Bristol Channel (Bristol, UK) was used for the research, and recycled plastic procured through the shredding process conducted at the University of South Wales was used. Table 1, Table 2 and Table 3 show the physical properties of cement, chemical properties of cement, and physical properties of aggregates and sand, respectively. Figure 1 shows the particle size distribution of the recycled plastics.

## 3. Methodology

### 3.1. Mix Design

The control mix served as a binder with a sand-to-aggregate ratio of 1:2:3. This control mix was formulated with Portland cement with a density of 340 kg/m^3^ and a water–cement ratio of 0.55. Typically, this control mix is classified as C30 concrete. Modifications were made to the control mix to assess the sand replacement potential of recycled plastic. Specifically, the sand content was reduced by up to 20% in volume percentage, and the removed portion of sand was replaced with recycled plastic, as outlined in Table 4. The first mix, referred to as JO1, is the control mix. For the second mix (JO2), the fine aggregate in the control concrete was replaced with 5% recycled plastic. In the third mix (JO3), the fine aggregate in the control concrete mix was replaced with 10% recycled plastic. For the fourth mix (JO4), the fine aggregate in the control concrete was replaced with 15% recycled plastic. In the fifth mix (JO5), the fine aggregate in the control concrete was replaced with 20% recycled plastic. All the replacement was carried out by volume. To replace plastic with fine aggregate in terms of volume percentage, it was necessary to determine the corresponding weight required for the replacement. To achieve this, a correction factor was taken into account. This correction factor was calculated by dividing the weight of the plastic in a specific container by the weight of sand needed to fill that container. This methodology allowed for an accurate conversion from volume percentages of plastic to their corresponding weight equivalents in the form of fine aggregate. Each mix incorporated 3.3 kg of water to maintain the specified water–cement ratio of 0.55. These variations allowed for the assessment of the impact of different levels of sand replacement with recycled plastic on the properties and performance of the concrete mixes.

### 3.2. Specimen Preparation

The materials used for the mix comprised cement, recycled plastic, sand, 10 mm aggregate, 20 mm aggregate, and water. Precise quantities of these materials for various mixes were batched by weight and stored separately in accordance with BS EN 206:2013+A2:2021 [12] to produce 10 cubes and 2 cylindrical specimens for each mix. A mechanized automatic mixer was employed to create the concrete mixtures. The ingredients for each mix were individually mixed within the automatic concrete mixer. To achieve homogeneity, the coarse aggregate, sand, recycled plastic, and cement were thoroughly mixed for a duration of two minutes. Subsequently, the measured water was added slowly into the drum, and mixing continued until a uniform concrete mixture was attained. The workability of the fresh concrete was then assessed by conducting a slump and compaction index test in accordance with BS EN 12350-2:2019 [13] and BS EN 12350-4:2019 [14], respectively. The hardened specimen preparation process, curing, and testing were carried out according to BS EN 12390-2:2019 [15], BS EN 12390-3:2019 [16], and BS EN 12390-6:2009 [16]. Lastly, the freeze and thaw investigation was carried out in accordance with PD CEN/TS 12390-9:2016 [17].

## 4. Results and Discussions

### 4.1. Consistency of Fresh Concrete

**Figure 2:**
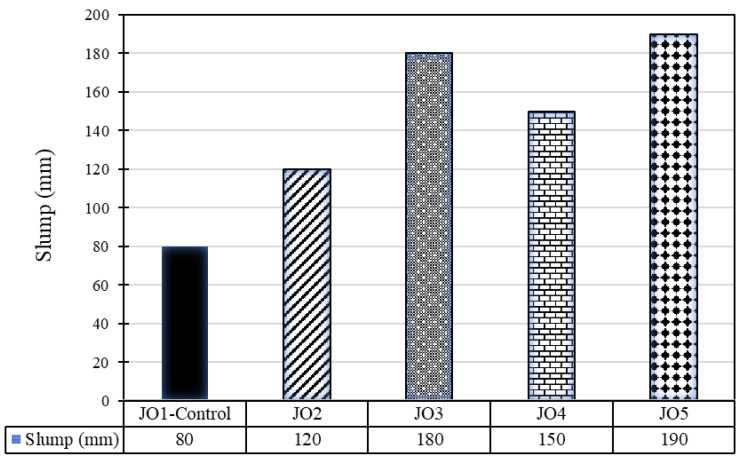
Consistency of concrete mixes measured—slump test.

Figure 2 shows the consistency of fresh concrete (slump test). The control mix achieved the lowest slump value of 80 mm, while Mix JO2 with 5% sand replacement with recycled plastic achieved the second-lowest slump value of 120 mm. However, Mix JO5 produced the highest slump, with a slump of 190 mm, while Mix JO3 produced the second-highest slump value (180 mm). Generally, the trend of continuous increase in slump values was consistent across the spectrum of mix combinations, except for one anomaly in Mix JO4 that displayed a lower slump than JO3, which is beyond the linear trend line of the other mixes (see Figure 2). The control mix, designated as JO1, had a slump value of 80 mm. This value suggests that the control mix exhibited relatively low workability and stiffness, which could be suitable for specific construction applications requiring minimal flow. The results indicate that as the percentage of sand replaced with recycled plastic increases, the slump value also increases. The addition of recycled plastic to the concrete mix led to a notable increase in workability and flowability.

**Figure 3:**
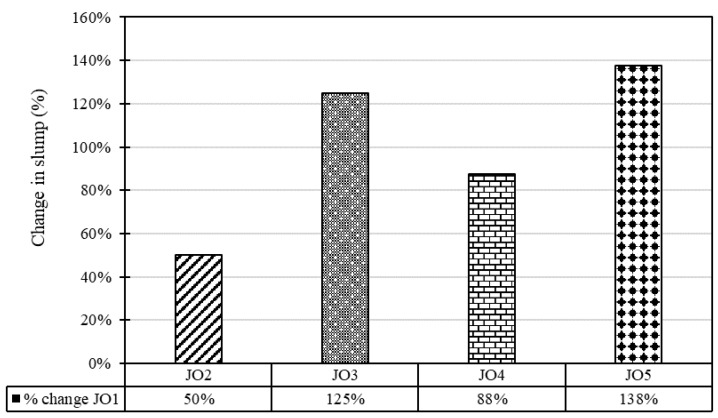
Percentage change in slump.

Figure 3 details the changing percentages of slump values with reference to the slump value of JO1—control mix. All the values are positive and range from 50% to 138%. The maximum changing percentage was observed in JO5, with a percentage of 138, and the least was for JO2, with a percentage of 50.

The change in the percentage of slump value compared to the reference mix varies from 50% (JO2) to 138% (JO5). The increased slump values in the blended mixes (JO2, JO3, JO4, JO5) compared to the control mix (JO1), suggesting that incorporating recycled plastic improved the workability properties of the concrete. This is consistent with the typical behavior of waste plastics, which can act as water reducers and lubricants in mixtures. Higher slump values indicate that the concrete mix becomes more flowable, easier to place, and compact [3]. This can be advantageous in construction scenarios requiring a more fluid mixture for proper consolidation and finishing. Hence, it is essential to determine the optimal percentage of recycled plastic replacement to achieve the desired workability without exceeding the practical limits [18].

### 4.2. Density of Hardened Concrete

**Figure 4:**
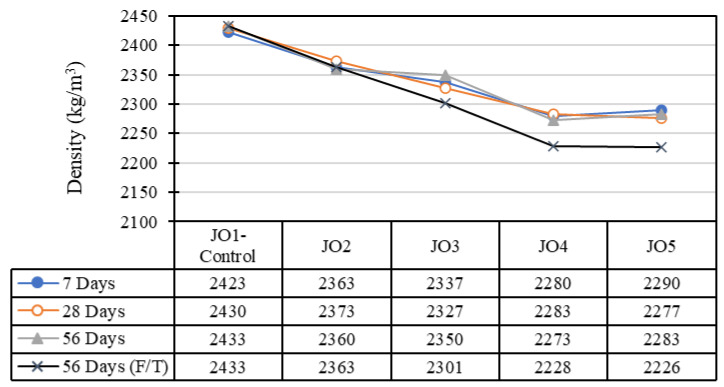
Density variation of concrete cube specimens (note: F/T = freezing and thawing).

**Figure 5:**
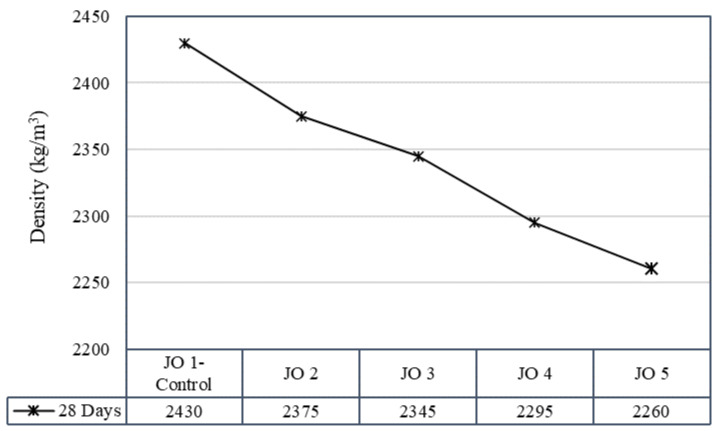
Density variation of cylindrical concrete specimens.

Concrete density is a critical factor influencing various aspects of concrete performance, including strength, durability, porosity, and permeability. Figure 4 shows the density variation of the developed concrete specimens. Observation showed that the density of concrete was reduced after the direct inclusion of recycled plastic in concrete mixes. Also, there was a visible decreasing trend in the density of the mixes, suggesting that some mixes may have experienced a reduction in density over time with the replacement of sand with recycled plastic. In both the cube specimens produced for the compressive test and cylindrical specimens cast for the tensile test, the control mix (JO1) generally achieved the highest density, while Mix JO5 had the lowest density. The density values for cylindrical specimens displayed a range of values, and a decreasing trend was observed in densities for the mixes at the end of 28 days of curing (Figure 5). The results suggest that the decrease in the density of specimens with an increase in the percentage of recycled plastic in the mix is primarily due to the lower density of plastic particles compared to natural sand [4,19]. Since plastic typically has a lower density than natural sand, introducing this less dense material into the concrete mix reduces the overall density of the concrete.

In addition to the lower density of plastic particles, the incompatibility of plastic and the cement mix is another significant reason for the reduced density. The interface between the plastic particles and the cement mix may be less effective than the sand–cement interface, resulting in more voids. Furthermore, irregularities in the size and shape of plastic particles can also contribute to the lower densities of concrete mixes. Larger or irregularly shaped particles may not pack as efficiently as sand, leading to a lower overall density.

### 4.3. Concrete Strength Development

#### 4.3.1. Without Subjecting Specimens to Freezing and Thawing

**Figure 6:**
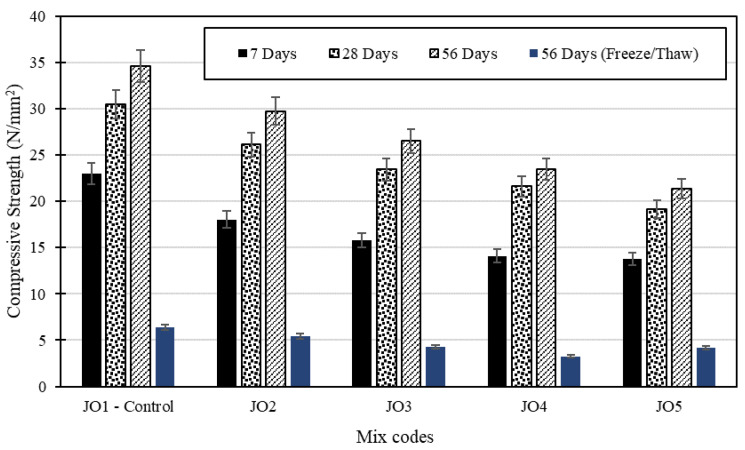
Variation in the compressive strength of the design mixes.

**Figure 7:**
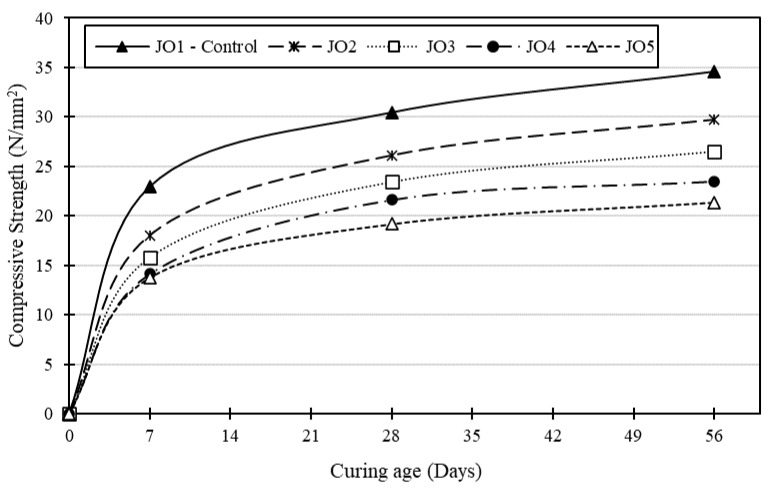
Compressive strength development in the design mixes with age.

Compressive strength determines how long a material can bear an applied load before failure, which is the most crucial aspect of the engineering properties of concrete. Depending on the design strength and the purpose of the structure, compressive strength typically ranges from 15 MPa to 40 MPa, though industrial and commercial constructions require a higher compressive strength. Figure 6 and Figure 7 show the variation in compressive strength (CS) development for all the concrete mixes.

Generally, observation showed that the control mix (JO1) consistently achieved the highest compressive strength values, and there was a significant increase in strength with time for all mixes across the 7-, 28-, and 56-day curing periods. However, Mix JO5 consistently had the lowest compressive strength, and there was a clear decreasing trend in strength with an increase in the percentage of recycled plastic in the mixes. Overall, Mix JO5 experienced the most substantial reduction in compressive strength compared to the control mix, with values ranging from −37% to −40%. Additionally, the early-stage test results (7 days) showed a higher sensitivity to the presence of recycled plastic, while the influence diminished with more extended curing periods (28 days and 56 days). This suggests that the influence of recycled plastic on compressive strength is more pronounced in the early stages of curing (7 days). This implies that the impact of recycled plastic on compressive strength becomes less significant as the curing period extends beyond 7 days.

In addition, there can be multiple reasons for the decrease in compressive strength as the percentage of recycled plastic replacing sand increases. One of the significant factors could be the weaker bonding between the mixed ingredients in the concretes made with recycled plastics. Plastic particles may not form strong physical or chemical bonds with the cement matrix, resulting in weaker adhesion [20,21].

#### 4.3.2. Subjecting Specimens to Freezing and Thawing

**Figure 8:**
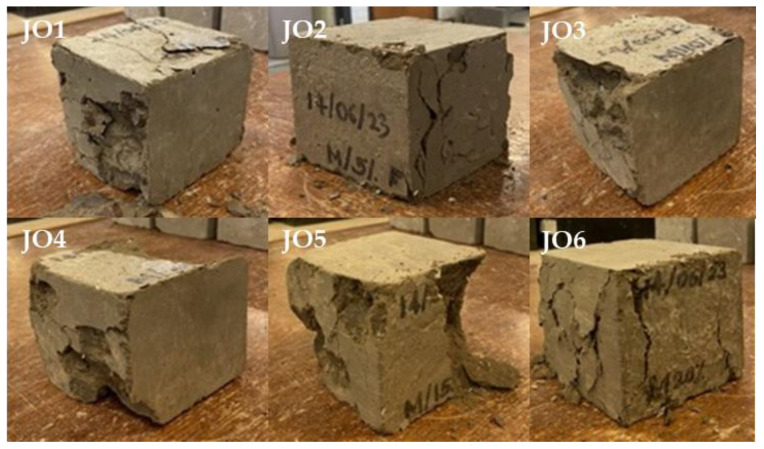
Performance of concrete cube specimens subjected to freeze and thaw cycles after Compressive Strength test.

This observation underscores the adverse effects of freezing and thawing cycles on the structural integrity and mechanical properties of the specimens, resulting in increased brittleness and a higher likelihood of failure when subjected to compressive forces. Despite the relatively minor effects on weight change and crack growth observed during the freezing and thawing cycles, the results revealed a significant decrease in the compressive strength of specimens subjected to these cycles. Compared to the normal specimens, the compressive strength of specimens subjected to freezing and thawing cycles exhibited a notable decrease in strength, amounting to approximately 80% (see Figure 6). This highlights the importance of assessing compressive strength as a critical parameter in evaluating the performance and durability of concrete under freezing and thawing conditions. In addition, the cubes subjected to freezing and thawing failed catastrophically (see Figure 8). The freezing and thawing test results revealed that while most specimens maintained their weight and structural integrity, two samples from Mix JO4 and one sample from Mix JO5 subjected to freezing and thawing exhibited a significant decrease in compressive strength and failed catastrophically during testing. This suggests that the concrete mixes, particularly those with recycled plastic, may have reduced durability when exposed to freezing and thawing conditions. However, further investigation and optimization may be required to enhance their resistance to freeze–thaw cycles.

The brittle nature and significant compressive strength decrease observed in concrete cube specimens made with part of the sand replaced by plastic particles and subjected to freezing and thawing can be attributed to several factors (Figure 8). Plastic particles have different material properties compared to sand. They are typically less dense and have lower mechanical strength. When mixed with concrete, they may not bond well with the cement paste, leading to weak interfaces and reduced cohesion within the concrete matrix. The use of plastic particles as a partial replacement for sand can disrupt the formation of a strong cementitious matrix. This can result in reduced cohesion between the concrete’s components, leading to weaker interparticle bonds and a more brittle nature. When concrete is subjected to freezing and thawing cycles, the water trapped within the concrete can expand as it freezes and contract as it thaws. This expansion and contraction can create internal stresses in the concrete. If the concrete is not well bonded and has weak interfaces due to the presence of plastic particles, it becomes more susceptible to cracking and spalling during freeze–thaw cycles. Moreover, plastic particles have different thermal expansion coefficients compared to sand and the cement matrix. During freezing and thawing cycles, the differential expansion and contraction of these materials can create internal stresses, leading to microcracks and weakening of the concrete. Further, plastic particles are generally hydrophobic and do not absorb or retain moisture like sand [21]. This can lead to localized variations in moisture content within the concrete, making it more vulnerable to freeze–thaw damage as water within the concrete freezes and thaws at different rates. Also, the replacement of sand with plastic particles may result in a decrease in the overall aggregate volume within the concrete [22,23]. This can affect the concrete’s ability to distribute loads and lead to more brittle behavior. Some plastic materials may not be chemically compatible with the alkaline environment of concrete, potentially leading to the degradation of the plastic particles over time. This degradation can further weaken the concrete and make it more prone to failure during freeze–thaw cycles.

### 4.4. Tensile Splitting Strength of Concrete

Tensile strength is the ability of concrete to withstand stress during load application without fracturing or cracking. Concrete’s tensile strength is approximately 10% of its compressive strength. Concrete’s tensile strength is notably lower than its compressive strength, making it prone to cracking. To assess how concrete behaves under tension, the split tensile strength of concrete is typically determined, as it is challenging to test concrete’s tensile strength directly. Applying true axial stress under direct tension is nearly impossible, leading to the examination of concrete’s tensile behavior through indirect testing methods. The split tensile test serves as an effective indirect means of evaluating concrete’s tensile strength. In this test, a standard cylindrical specimen is laid horizontally, and the force is applied on the cylinder radially on the surface, which causes the formation of a vertical crack in the specimen along its diameter.

**Figure 9:**
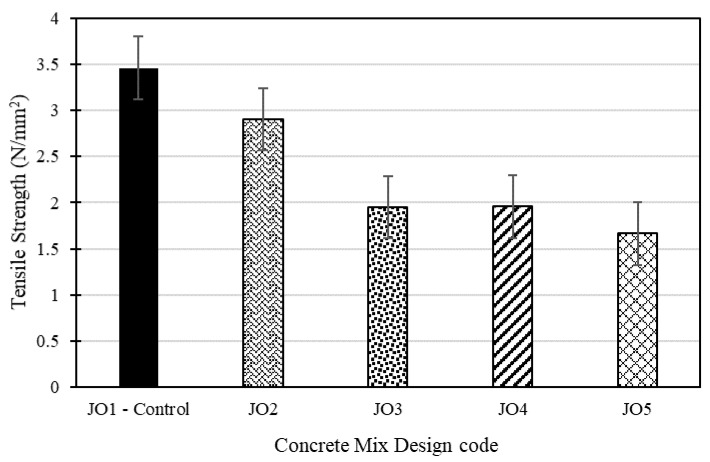
Variation in tensile splitting test results for the design mixes.

Figure 9 shows the splitting test results of the developed concrete mixes, where the control mix exhibits the highest resistance to tensile forces, implying that the presence of recycled plastic had a relatively minor impact on the tensile split strength of Mix JO2. In summary, Mix JO1 (control mix) demonstrated the highest tensile split strength at 28 days, whereas Mix JO5 exhibited the lowest tensile strength. In JO5, tensile strength was 52% lower than that of the control mix (see Figure 9). Recycled plastics typically exhibit lower tensile strength when compared to natural sand. When recycled plastics replace sand, which is a strong and dense material, the concrete mix is introduced to a weaker material in terms of tensile strength. This inherent weakness of plastics contributes to an overall reduction in the tensile strength of the concrete. Furthermore, the presence of recycled plastic particles in the mix can diminish the overall cohesion of the concrete. Cohesion is critical for resisting tensile forces, and any decrease in cohesion can result in a reduced tensile strength of the concrete. Brittleness and the incompatibility of recycled plastic with the cement matrix can also be additional factors contributing to the reduction in tensile strength with an increase in the percentage of recycled plastic used in the mix. These factors collectively emphasize the importance of carefully considering the use of recycled plastics in concrete mixes, particularly in applications where tensile strength is a critical requirement.

Furthermore, it was observed that there was a uniform distribution of recycled plastic particles (PPs) throughout the mix after splitting the cylinders into two parts. This observation indicates that the recycled plastic particles are evenly dispersed within the mix, and there is no evidence of clogging or clumping. To maintain this uniformity, it is essential to carefully calculate the proportion of plastic in relation to other ingredients in the concrete mix. Additionally, the workability and consistency of the concrete mix play a crucial role in achieving this uniform distribution of plastic particles within the mix. Non-uniform distribution can result in variations in strength and other properties, making the concrete less predictable and reliable for structural applications. Therefore, the uniform distribution of plastic particles in concrete made by replacing sand with recycled plastic is a positive property contributing to its overall performance and reliability.

Future studies can be performed by adding other reinforcing materials, such as steel or synthetic fibers, to the concrete mix to compensate for the reduction in compressive strength due to plastic. These fibers can enhance the tensile and flexural strength of concrete. Making changes to the water–cement ratio, aggregate type, and mixing procedure can indeed help adjust and optimize the workability of concrete when incorporating waste plastics. These adjustments can help isolate the specific effect of plastic on the concrete mixture and allow for more precise control over the mix’s properties. Increasing the sample size and the number of replicates for each percentage of plastic replacement is also a valuable strategy to enhance the statistical power of research results. A larger sample size provides a more robust dataset, which can lead to more reliable and generalizable findings. It can help researchers draw more definitive conclusions about the impact of waste plastics on concrete properties. By implementing these measures and conducting well-designed experiments, researchers can better understand how to effectively use waste plastics in concrete while maintaining the desired workability and performance characteristics. These methods will be utilized if there is a chance to conduct the study in order to obtain more effective results.

## 5. Conclusions

The findings of this study indicated that there was a possible change in the consistency and strength of concrete when sand was replaced with recycled plastic. This investigation yielded the following conclusions:

1. The workability improved when sand was replaced with recycled plastic. This improvement resulted from the hydrophobic nature of the plastic particles, requiring less water to wet their surface, ultimately enhancing workability. Additionally, the inclusion of plastic particles improved the rheological properties and uniformity of the concrete matrix due to their hydrophobic characteristics.
2. A decreasing trend in the density of hardened concrete with an increase in recycled plastic that replaced sand was observed, which was attributed to the lowered density and incompatibility of plastic particles.
3. Mixes with a lower percentage, i.e., 5%, of replacement of sand with recycled plastic exhibited relatively high compressive strength.
4. Higher replacement percentages of sand with recycled plastic, such as 15% and 20%, resulted in reduced compressive strength. This reduction could be attributed to decreased density, weaker bonding, incompatibility, and non-uniform properties of the recycled plastic particles.
5. A relatively minor impact of recycled plastic on tensile split strength was observed in the tested samples. The decrease in tensile strength may have occurred due to the lower cohesion of PPs, brittleness, and incompatibility.
6. A uniform distribution of plastic particles was observed during the tensile splitting test, with no clumping or uneven distribution. This ensured that the concrete mix maintained its desired workability and consistency. This is a positive property that contributes to its overall performance and reliability.
7. Concrete made by replacing sand with recycled plastic showed poor resistance to freezing and thawing cycles. Compressive strength decreased by around 80% compared to samples not subjected to freezing and thawing. Simultaneously, these samples exhibited brittle properties. Possible reasons for this behavior include differential thermal expansion between recycled plastic and concrete, reduced bonding strength between recycled plastic particles and the concrete matrix compared to sand and cement paste, and the incompatibility of recycled plastic particles with the cement matrix.

## Figures and Tables

**Table 1:** Physical properties of PC [9].

Properties	PC
Insoluble residue	0.5
Bulk density (kg/m^3^)	1400
Specific Gravity	3.15
Blaine fineness (m^2^/kg)	365
Loss of ignition (%)	0.8
Color	Grey
Glass content	-

**Table 2:** Chemical compositions of PC [10].

Oxide	%
Calcium oxide (CaO)	60
Silicon dioxide (SiO_2_)	20
Aluminium oxide (Al_2_O_3_)	6
Magnesium oxide (MgO)	4.21
Iron oxide (Fe_2_O_3_)	3
Manganese oxide (MnO)	0.03–1.11
Sulphide (S_2_)	_
Sulfur trioxide (SO_3_)	2.3
Alkalis	_

**Table 3:** Physical properties of aggregates [11].

Property	10 mm Coarse Aggregate	20 mm Coarse Aggregate	Sand
Water absorption (%)	1.5	12.8	0.85
Saturated density (Mg/m^3^)	2.68	1.33	2.82
Dry density (Mg/m^3^)	2.57	1.42	2.71
Shape index (%)	12	32	-
Impact value (%)	18	12	-
Flakiness index (%)	23	37	-

**Table 4:** Mix design and material quantities used to produce concrete test specimens.

Mix Code	PC (kg)	Water (kg)	Coarse Aggregate (kg)		Sand (kg)	Recycled Plastic	Plastic in Sand%
			10 mm	20 mm			
JO1—Control	6	3.3	6	12	12	_	_
JO2	6	3.3	6	12	11.4	0.12	5
JO3	6	3.3	6	12	10.8	0.24	10
JO4	6	3.3	6	12	10.2	0.36	15
JO5	6	3.3	6	12	9.6	0.48	20

## Data Availability

Data are contained within the article.
